# Novel image-free arc detection method for pantograph–catenary systems based on direct DWT–ANN signal analysis

**DOI:** 10.1038/s41598-026-56456-y

**Published:** 2026-06-19

**Authors:** Mohamed S. Elbelkasi, Ebrahim A. Badran, Nagy I. Elkalashy, Mansour H. Abdel-Rahman

**Affiliations:** 1 Electrical Engineering Department, Faculty of Engineering, Mansoura University, 35516 Mansoura, Egypt; 2 Mansoura Higher Institute of Engineering and Technology (Mansoura Collage), Mansoura, Egypt; 3 Electrical Engineering Department, Faculty of Engineering, Menoufia University, Shebin Elkom, 32511 Egypt; 4 Faculty of Engineering, Mansoura National University, Gamasa, Egypt

**Keywords:** Image-free arc detection, DWT–ANN hybrid framework, Pantograph–catenary system, Short length Arc modeling, Series arc detection, Real-time pantograph arc monitoring, Engineering, Mathematics and computing

## Abstract

Arc faults in pantograph catenary systems pose a significant threat to the reliability, safety, and efficiency of the electric railways. However, Conventional approaches relying on image processing and deep learning are hindered in real-time applications due to computational delays exceeding the arc time constant. Therefore, this study proposes a novel image-free arc detection method that directly analyzes measured traction current signals using DWT–ANN (Discrete Wavelet Transform-Artificial Neural Networks). The significance of this work lies in its ability to extract transient arc features directly from the traction current waveform, providing a computationally efficient solution for real-time monitoring without the need for additional sensing modalities. Various mother wavelet families are evaluated, and specific detail levels are identified as the most informative for capturing arc transients. Among these, Daubechies db9 and Symlet sym8 showed the highest discriminative performance and were used as inputs to a compact ANN structure. The network, trained with normalized feature data, achieved a high regression coefficient, indicating excellent classification accuracy. Additionally, algorithm robustness was validated by training the neural network on db4-extracted features and testing it across all accepted wavelets, with consistent detection performance. The results confirm the robustness and practicality of the proposed DWT-ANN framework for real-time arc detection in railway systems.

## Introduction

The pantograph-catenary system plays a vital role in high-speed railway operations by ensuring continuous and efficient energy transfer from the overhead contact line to the train. However, this system is prone to faults, particularly electrical arc faults, which arise due to intermittent discharges between the pantograph and the catenary. These arc faults not only compromise system reliability and safety but can also lead to operational disruptions. Their transient and non-stationary nature presents significant challenges for detection and diagnosis using conventional signal analysis methods^1^-^2^.

For arc modelling applications, the selection of an arc model depends on the application, such as transient analysis, insulation coordination, or breaker design, and the desired accuracy. Model parameters like its time constant, stationary dissipated power, and stationary arc voltage are usually obtained through experiments or optimized for specific breakers and quenching media^3–5^. The arc models and simulations can participate in evaluating the arc performance, including the arc reignition. Arc reignition typically occurs after each current zero-crossing, resulting in waveforms that are highly non-stationary and stochastic. The arc reignitions produce transients in the power systems that can be exploited to find fingerprints of the arc occurrence using the digital signal processing techniques^6^.

Traditional signal processing techniques, such as the Fast Fourier Transform (FFT), which assume signal stationarity, are not effective for timely and accurate arc detection in such cases. Consequently, the Discrete Wavelet Transform (DWT) has gained prominence as a more appropriate method for analyzing the complex time-frequency characteristics of arc signals. DWT decomposes signals into multiple frequency bands, providing localized time and frequency information, which is essential for identifying transient events such as arcs in pantograph-catenary systems^7^.

In addition to its time-frequency localization capabilities, DWT is effective in distinguishing arc-related signal components from background noise. This makes it highly suitable for enhancing the visibility of arc features embedded within noisy traction current or voltage signals^8–10^. As a result, DWT has become a central tool in modern arc fault detection frameworks for electric railway systems. The integration of DWT with artificial neural networks (NNs) has further improved the accuracy and robustness of arc fault detection. Neural networks are machine learning models capable of learning complex, nonlinear patterns from data. When DWT is used to extract discriminative features from transient signals, these features can be used as inputs to NNs for classification. This hybrid DWT-NN approach effectively combines the strengths of both techniques—DWT’s signal decomposition and NN’s pattern recognition capabilities, resulting in superior performance for identifying arc events^11^^-^^12^.

From the above-discussed literature, the applications of wavelet transform and neural networks on the arc detection of the pantograph systems were considered for image processing. However, such detection systems are not applied or evaluated for the electrical features of series arcing faults inherent in the electrical quantities, such as the current and voltage waveforms associated with the pantograph-catenary systems. Furthermore, implementing the image processing approach in according with deep learning methods provide a high challenge against real-time implementation of arc detection in pantograph catenary systems. As the DWT-NN framework has demonstrated strong adaptability to the dynamic conditions typical of dynamic arc behavior, such a system is used to track and respond to variations, and its utility in real-world deployment is enhanced for railway fault monitoring^2^, ^12^^-^^18^.

The integration of advanced computational frameworks has significantly enhanced the performance of industrial fault diagnosis systems. Recent studies have introduced explainable diagnostic approaches based on burst-informed acoustic emission analysis, enabling improved interpretation of failure mechanisms^19^. In parallel, transformer-based models combined with semi-supervised learning and uncertainty quantification have demonstrated high accuracy in tool condition monitoring under limited labeled data scenarios^20^. Furthermore, multi-sensor observer-based residual learning methods have shown strong robustness in fault diagnosis under varying operating conditions^21^. Despite these advancements, many of these approaches rely on computationally demanding architectures or image-based representations, which may limit their applicability in real-time engineering systems. In contrast, the proposed method adopts a direct electric signal-based, image-free framework using DWT for feature extraction combined with a lightweight ANN model, enabling efficient and reliable real-time arc detection in pantograph–catenary systems.

In addition to these approaches, recent studies have further explored advanced data-driven methods for fault diagnosis under varying operating conditions. For instance, multistage transfer learning frameworks have been proposed to improve feature adaptability and enhance performance in data-scarce scenarios^22^. In addition, physics-guided and hybrid deep learning models have demonstrated improved robustness and interpretability in industrial fault detection tasks^23^. Moreover, optimized transfer learning strategies and temporal deep learning architectures have been applied to improve feature representation and generalization in machine condition monitoring systems^24,25^. These developments highlight the increasing importance of robust feature extraction and model generalization in modern fault diagnosis frameworks.

Recent research has explored non-vision-based approaches to overcome the limitations of image-based arc detection under varying environmental conditions. For instance^26^, proposed a dual-modal sound–optic fusion method, while^27^ investigated electrical transient characteristics during current-zero periods for arc detection. In addition, deep learning models such as^28^ have demonstrated high accuracy in detecting series AC arcs using current signals. However, these methods are either designed for general power systems or rely on multi-sensor configurations and relatively complex processing schemes. In contrast, the proposed method focuses on pantograph–catenary systems and directly utilizes traction current signals. By employing DWT for feature extraction and a lightweight ANN for classification, the proposed framework provides a computationally efficient and practically deployable solution suitable for real-time railway applications.

Beyond fault detection, recent research in intelligent maintenance has increasingly focused on prognostics and health management (PHM). Advances in this area have introduced data-driven frameworks for remaining useful life (RUL) prediction, including source-free domain adaptation^29^ to improve model generalization across different operating conditions without requiring source data. In addition, interpretable deep learning models have been developed to better characterize system degradation and quantify uncertainty in prediction^30^. In this context, the proposed DWT-ANN method utilizes wavelet-based features that capture the transient characteristics of arc events while maintaining computational efficiency. This provides a practical and interpretable signal-based approach suitable for reliable monitoring in pantograph–catenary systems.

In this paper, arc feature extraction and detection are uniquely performed by directly processing measured electrical quantities—specifically, the traction system current—rather than relying on digital image processing techniques, representing a direct and application-specific use of traction current signals for arc detection in pantograph–catenary systems. Therefore, the arc associated with the pantograph-catenary system is represented using ATP Draw as a series dynamic element. The corresponding transients due to arc reignitions are extracted using the DWT algorithms. The extracted arc transient features are evaluated concerning different detail levels and different mother wavelet families, such as Daubechies family, the Bio-Orthogonals family, the Coiflets family, and the Symlet family. Accordingly, the best detail levels and mother wavelets are estimated and preprocessed to calculate the absolute sum of a sliding window of power cycle size. These processed signals are normalized and used as input to the neural network for the arc detection applications. The results confirm the efficiency of the proposed system for arc feature extraction and detection using the measured electrical quantity, which is the pantograph-catenary current.

The paper flow is that the arc evolution concerning the arcs associated with circuit breakers, arcing faults, either long or short arc lengths, and the pantograph-catenary system is discussed in section II, the arc feature extraction accomplished using the DWT is in section III, section IV includes the proposed approach for detecting the pantograph arcs using neural networks, and section V presents the conclusions.

## Arc modelling

Arc models are crucial for understanding and mitigating the effects of arc in several applications, such as Circuit Breaker (CB) and its Quenching Media, as well as the associated arcs with transmission lines (T.L) and distribution systems. These models help to simulate the behavior of arcs, which are complex due to their nonlinear and dynamic nature. The dynamic resistance is the most representative of arc modelling as an electrical element. The dynamic arc resistance is due to the dynamic arc conductance that is estimated using the energy balance concept, which is the balancing between the input arc energy due to its current and the dissipated surrounding energy. Accordingly, the literature papers provide insights into various arc models, their applications, and advancements in arc fault modeling and detection. The general arc modeling equations are presented as a black box model, meaning they describe the arc’s electrical behavior without detailing the complex physical processes within the arc plasma. It is in the form^31^;

1 $$\:\frac{\boldsymbol{d}g}{\boldsymbol{d}\boldsymbol{t}}=\:\frac{1}{\boldsymbol{\tau\:}}\left(\:G-g\:\right)$$

where, g is the dynamic arc conductance, which is a time-varying conductance. The dynamic equation parameters are the arc time constant τ and the stationary arc conductance G. These parameters are variables on the other factors, depending on the applications and arc characteristics. Accordingly, several arc models are presented in the literature concerning the applications, as summarized in Table 1 and discussed as follows.

**Table 1:** Comparative overview of arc models^32–39^.

Application	Arc model	Stationary arc conductance G	Parameters Decleration
Circuit breakers	*Mayr*	$\:G=\:\frac{\:{i}^{2}}{\:\:p\:}$	$\:p$ : const
	Cassie	$\:G=\:\frac{\:{i}^{2}}{g\:\:{{U}_{st}}^{2}\:}$	$\:{U}_{st}$ : Const
	Habedank	*Mayr &* Cassie	
	*KEMA*	$\:\mathrm{G}=\:\frac{\:{i}^{2}}{p}$	$\:\mathrm{p}=\mathrm{max}\left(\:{U}_{o}\left|i\right|,\:{P}_{o}+\:{P}_{1}\:u\:i\right)$
Arcing Fault	Kizilcay	$\:G=\:\frac{\left|i\right|}{{U}_{st}}$	$\:{U}_{st}=\left(\:{u}_{o}+\:\left|i\right|r\:\right)l$
	Johns		$\:{U}_{st}={u}_{o}l$
	Short arc		$\:{U}_{st}$: const
Pantograph	Pantograph	$\:G=\:\frac{{i}^{2}}{{P}_{t}}$	$\:{P}_{t}=L\:{P}_{diss}$ $\:{P}_{diss}=0.8\:{K}_{1}\left(\upsilon\:+10\right)\sqrt{I}$

## Arc models in circuit breaker applications

The interruption of fault currents using circuit breakers relies on the effective quenching of the electric arc that forms between the separating contacts. Understanding and accurately modelling this arc is crucial for designing efficient and reliable circuit breakers. The behavior of the arc is highly non-linear and dynamic, influenced by the properties of the quenching medium (e.g., air, SF6, vacuum, oil) and the power system parameters. The most widely used and fundamental arc models are the Mayr model, the Cassie model, and their various combinations or modifications.

The Mayr model is well-suited for characterizing arc behavior near the current zero-crossing, where the current magnitude is minimal, and energy dissipation is predominantly governed by thermal conduction. It assumes that the arc’s cross-section is constant and that the arc energy is primarily lost through thermal conduction to the surrounding quenching medium. The arc conductance G in Eq. (1) becomes:

2 $$\:G=\:\frac{\:{\boldsymbol{i}}^{2}}{\:\boldsymbol{p}\:\:}$$

where i is the arc current, and p is the power dissipated by the arc, which is the steady-state cooling power of the arc.

The Cassie model is more appropriate for describing the arc behavior at high current levels, away from current zero. It assumes that the energy dissipation is mainly due to convection and that the arc voltage remains relatively constant. The expression for arc conductance G in Eq. (1) can be rewritten as:

3 $$\:G=\:\frac{\:{\boldsymbol{i}}^{2}}{g\:\:{{\boldsymbol{U}}_{\boldsymbol{s}\boldsymbol{t}}}^{2}\:}$$

where Ust is the steady-state arc voltage and it is called the stationary arc voltage. This is assumed to be constant under high current conditions.

The third arc model presented for the circuit breaker applications is Habedank’s Model, which integrates both Cassie and Mayr models to utilize their strengths across different arc conditions. It is typically combines their conductance in parallel or switches based on arc parameters. Where, total arc conductance g is a combination of Cassie g_c_ and Mayr gm conductance.

4 $$\:\frac{1}{\boldsymbol{g}}=\:\frac{1}{{\boldsymbol{g}}_{\boldsymbol{m}}}+\:\frac{1}{{\boldsymbol{g}}_{\boldsymbol{c}}}$$

where gc and gm are governed by their respective differential Eqs. (2) and (3), respectively. The advanced Cassie-Mayr models use switching or smooth transitions between modes, especially near or after the current zero for transient recovery voltage TRV analysis.

The fourth model is the KEMA model, which is an empirical model developed for simulating high-voltage circuit breaker arcs, particularly under short-circuit conditions. The KEMA Model consists of multiple modified Mayr models. From Equ (1), the arc conductance G is expressed as:

5 $$\:G=\:\frac{\:{\boldsymbol{i}}^{2}}{p}$$

6 $$\:\mathrm{p}=\mathrm{max}\left(\:{U}_{o}\left|i\right|,{P}_{o}+\:{P}_{1}\:u\:i\right)$$

where p is the power dissipated by the arc where P1 and Po are constants of cooling power, and U_o_ is the constant arc voltage in the high current area.

Other arc models exist for the circuit breaker applications, including Schwarz’s arc model, hydrodynamic models, and empirical models. These models vary in complexity and application, offering alternative methods for capturing arc behavior under different electrical and physical conditions.

## Models of arcs associated with power system faults

Transmission line arcing faults, typically classified as transient faults, are common in power systems but more difficult to detect than other transient events. To accurately capture the interaction between the arc and the system, efficient long arc models are essential^35–38^. Their dynamic behavior is generally modelled using normalized volt-ampere characteristics or power-based methods. Further applications of the arc models were for short-length arcs that are associated with high impedance faults in distribution networks^39^. For the arc faults either associated with the power transmission lines or distribution networks, the arc equation of the model is given as in Eq. (1), where the following formulation for the arc conductance G is defined as:

7 $$\:G=\:\frac{\left|i\right|}{{U}_{st}}$$

Two famous dynamic models for long arcs associated with the high voltage insulators have been introduced: the Kizilcay model^35,36^ and the Johns model^33^ developed to represent arc parameters for both primary and secondary arc phases over 380 kV insulation string. The Kizilcay model considers the stationary arc voltage in (7) by^36^.

8 $$\:{U}_{st}=\left(\:{u}_{o}+\:\left|i\right|r\:\right)l$$

where *u*_*o*_ is a constant voltage per arc length, *r* is the resistive component per arc length, and *l* is the time-dependent arc length. The Johns model^34^provides a method for evaluating the stationary arc conductance $\:G$ in (1) and then the stationary arc voltage in (8) is evaluated as:

9 $$\:{U}_{st}={u}_{o}l$$

For the distribution networks, the model describes short-length arcs with minimal spatial development, assuming a uniform voltage drop and quasi-stationary behavior^18^. Using the same arc dynamics in (1) and the arc conductance G in (7), the stationary arc voltage Ust is a constant value.

From the discussion in this subsection of arc modelling for power transmission lines and distribution networks, the associated arcs are in the air environment as a quenching medium. The appropriate formula of the stationary arc conductance is related to absolute arc current (|*i*|) over the stationary arc voltage *U*_*st*_, which is described differently for each model.

## Pantograph arcing

It is a recurrent phenomenon in high-speed rail systems, resulting from transient disconnections between the pantograph and the catenary wire. This arcing is fully exposed to the surrounding atmospheric environment, and its behavior is significantly influenced by parameters such as the train velocity and the pantograph–catenary gap.

For the pantograph arc model, the arc conductance G in Eq. (1) takes the form^1^:

10 $$\:G=\:\frac{{i}^{2}}{{P}_{t}}$$

11 $$\:{P}_{t}=L\:{P}_{diss}$$

12 $$\:{P}_{diss}=0.81\:{K}_{1}\left(\upsilon\:+10\right)\sqrt{I}$$

where, *I* is the rms value of current, *P*_*diss*_ is the dissipated power per unit length generated, *k*_1_ is the arc correlation coefficient of dissipated power, $\:\upsilon\:$ is the velocity (running speed of the train), and $\:{P}_{t}\:\mathrm{i}\mathrm{s}\:$the total dissipated power of the pantograph arcing.

## Discussions

From the arc modelling methods that were presented in the literature, most of the modeling algorithms depend on the black box model Eq. (1). The variable arc model parameters were presented with different declarations as aforementioned. For the pantograph arc consideration, the velocity is a factor affecting the arc parameters concerning Mayr representation. However, the accuracy of Cassie is better than the Mayr model as reported in^28^. This is expected as the arc is in the air as concluded in subsection II.B. Furthermore, the velocity time constant (the time to change the velocity) is a mechanical time, which is very high with respect to the arc time constant. Accordingly, the arc parameter G in Eq. (1) can be simplified to a short arc parameter that is |i|/Ust, where Ust is simply estimated from the arc voltage waveform that is at the instantaneous peak current. Consequently, it is considered 2.3 kV for the following results.

This simplification is based on preserving the dominant electrical characteristics of the arc relevant to transient detection while reducing model complexity. The adopted formulation follows the modified Mayr model, where the arc conductance evolves dynamically. For implementation efficiency, this is represented using the relation G = ∣i∣ / Ust, where Ust denotes the stable arc voltage. This approximation remains physically consistent, yielding conductance values within the same order of magnitude as those obtained from detailed arc models, and is in agreement with previously reported studies^1^. Consequently, this representation maintains the essential transient characteristics of the pantograph arc while significantly reducing computational complexity, making it suitable for system-level simulations and real-time signal analysis.

**Fig. 1:**
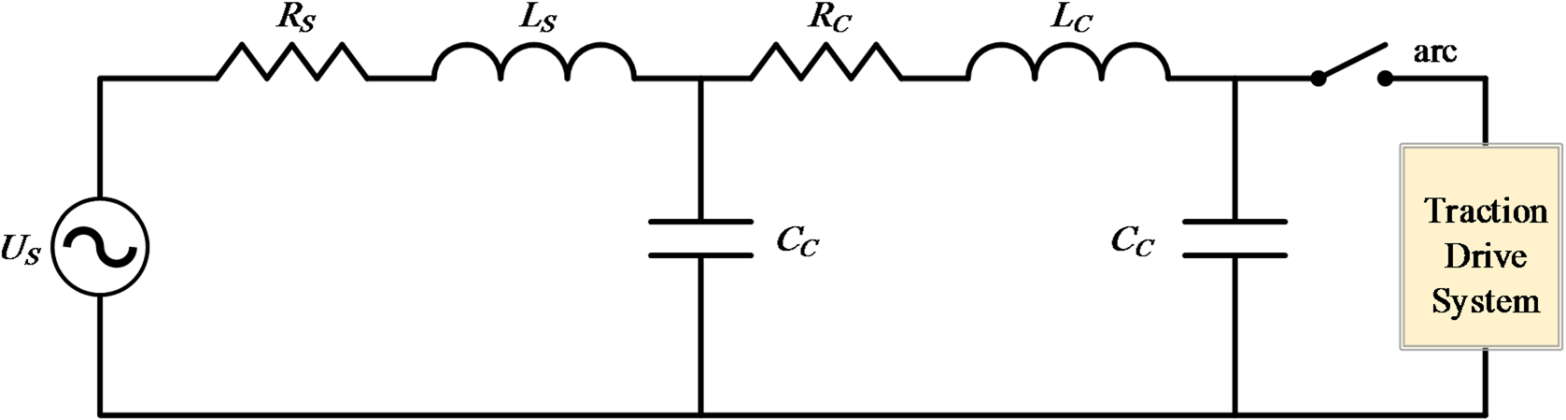
Traction equivalent circuit.

Figure1 shows the equivalent circuit of the traction system. The elements US, RS, and LS represent the equivalent voltage source, resistance, and inductance of the traction transformer, with respective values of 35.35 kV, 0.14 Ω, and 3.16 mH. The catenary wire is modelled as a π-type equivalent circuit consisting of RC, LC, and CC, which correspond to its equivalent resistance, inductance, and capacitance to ground. These are assigned values of 2.95 Ω, 23.5 mH, and 0.081 µF, respectively.

**Fig. 2:**
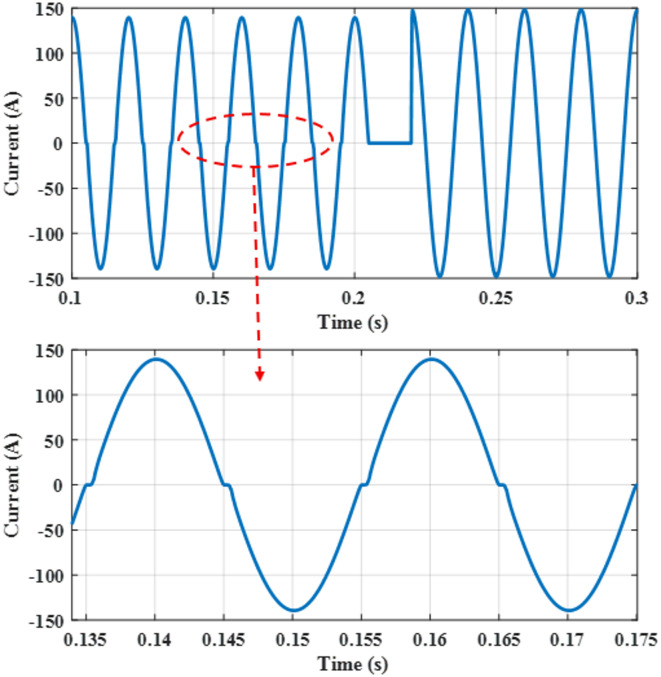
Enlarged view of system current waveform.

**Fig. 3:**
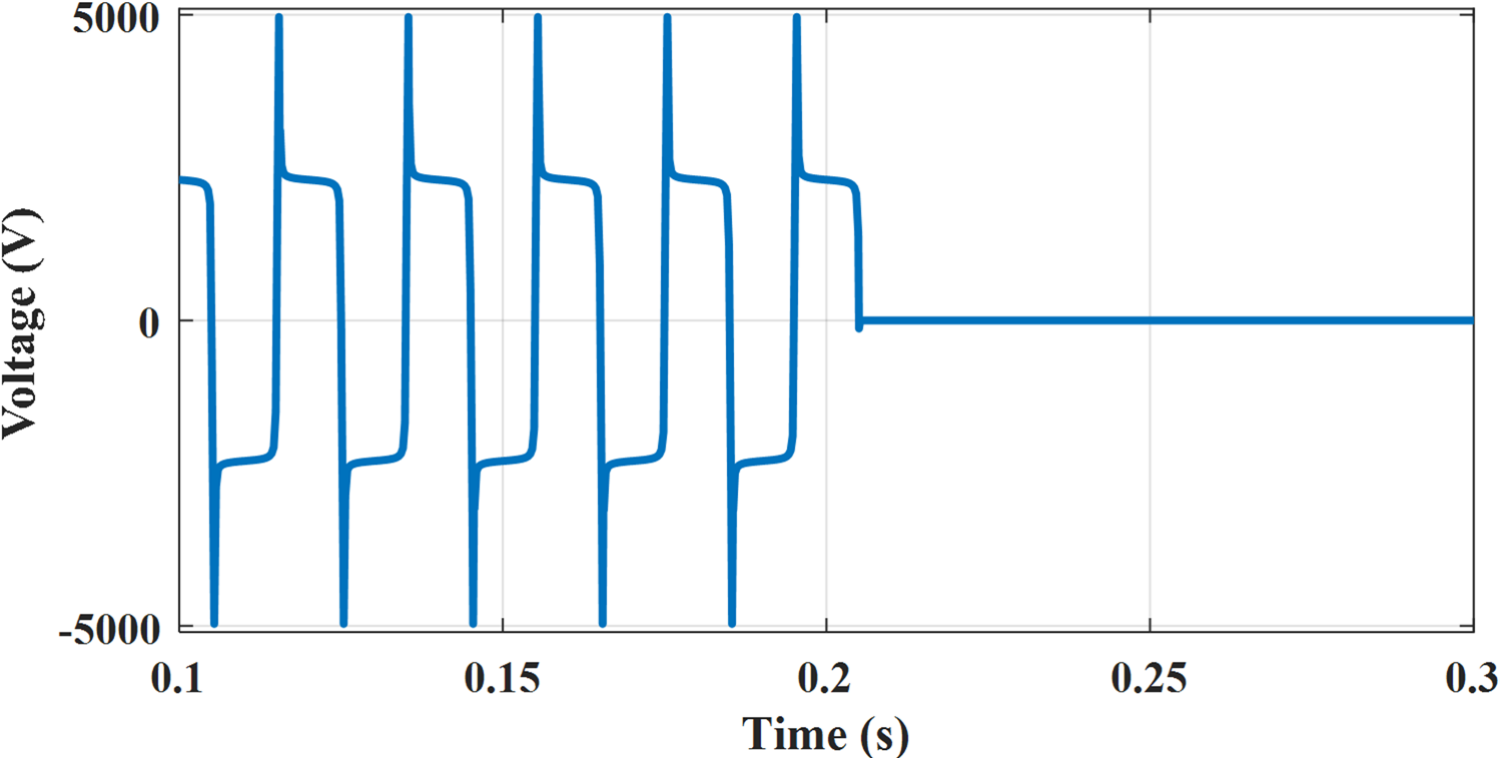
Arc voltage waveform.

**Fig. 4:**
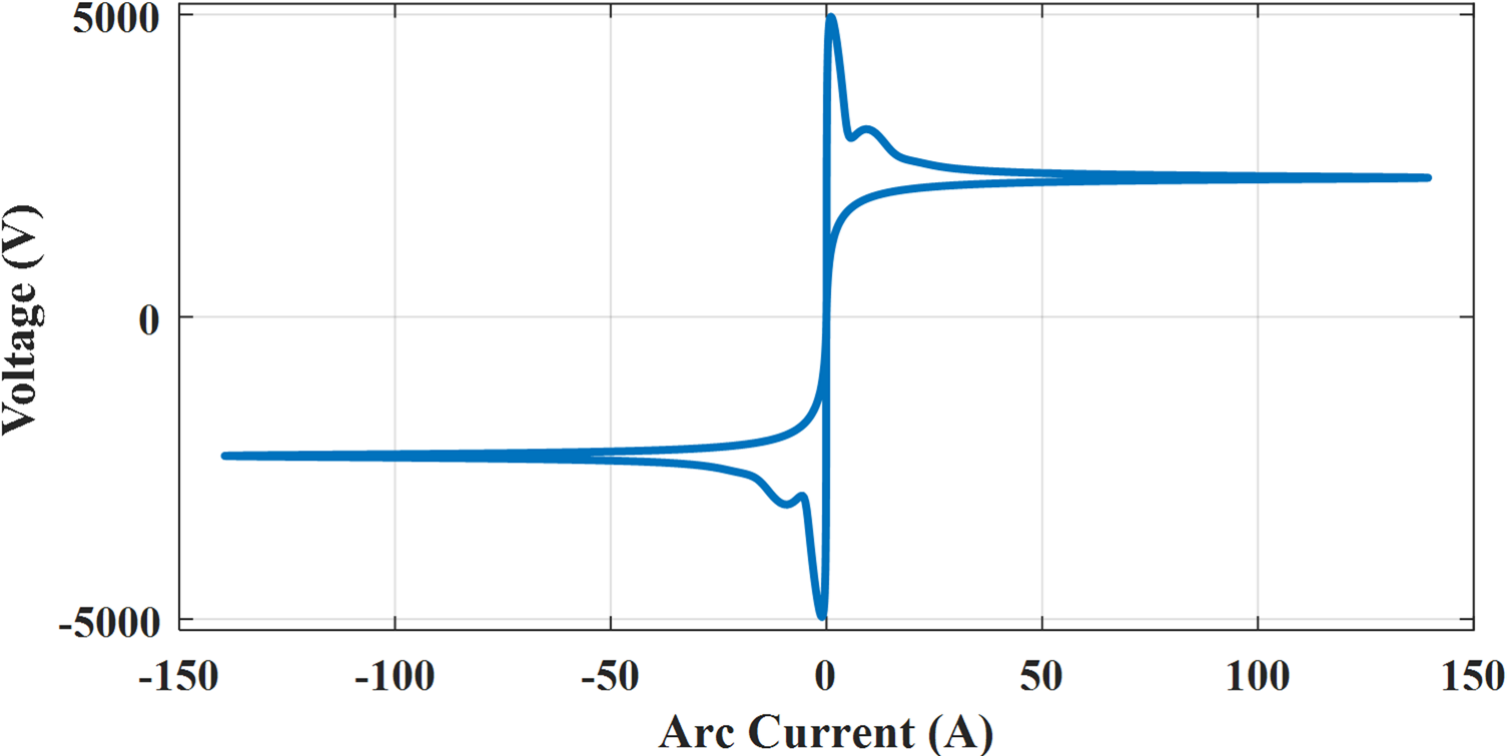
Arc characteristics.

By simulating using ATP Draw that is the user interface of the ATP program, the taken measurements are of the load current and arc voltages are presented in Figs. 2 and 3. As shown in the zoomed Fig. 2, there is an arc reignition repeated at the current zero crossing. The arc extinction is accomplished at 0.205 s and the normal load current without associated arc is restored at 0.22 s. From Fig. 3, the arc voltage distortion is shown, and the corresponding arc (v-i) characteristics are presented in Fig. 4, confirming the arc creation. This simulated system is considered for generating the series arcs associated with the pantograph catenary system and, therefore, testing the efficiency of DWT for extracting arc transients as in the following section.

**Fig. 5:**
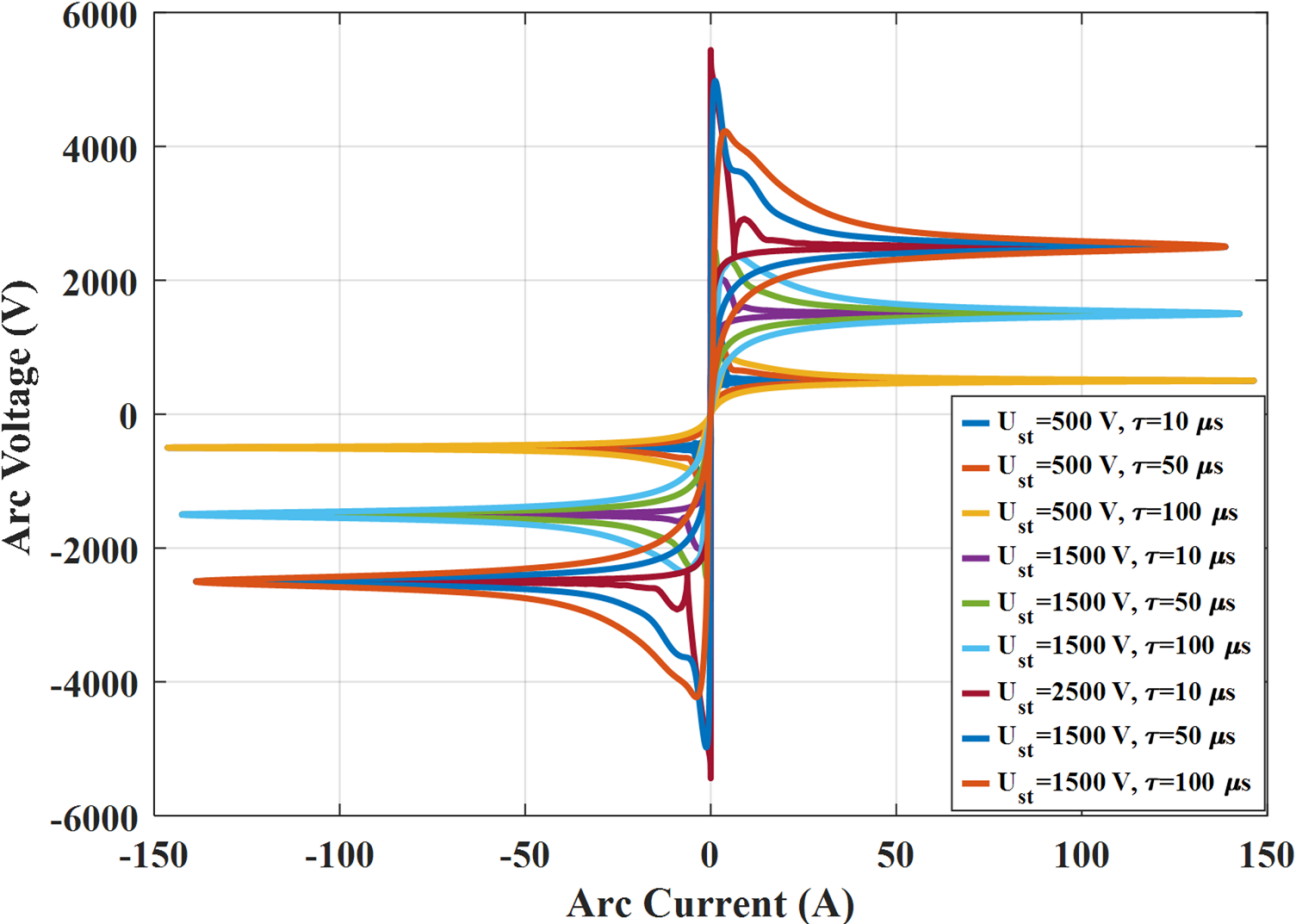
Arc voltage–current characteristics under different Ust and τ.

As further validation, Fig. 5 illustrates the arc voltage–current characteristics under different operating conditions. The results confirm that the adopted model preserves the nonlinear arc behavior and key transient features across varying parameter values, thereby supporting the validity of the simplified conductance representation.

## DWT-based arc feature extraction

The Discrete Wavelet Transform (DWT) is a powerful signal processing tool that decomposes a signal into time-localised frequency components by applying discrete scaling and translation to a single mother wavelet function. Mathematically, the DWT is expressed as^40^:

13 $$DWT_{\psi } f(m,k) = \frac{1}{{\sqrt {a_{o}^{m} } }}\sum\limits_{n} {x(n)\psi (\frac{{k - nb_{o} a_{o}^{m} }}{{a_{o}^{m} }})}$$

where *ψ*(.) is the mother wavelet that is discretely dilated and translated by *a*_*o*_^*m*^ and *nb*_*o*_*a*_*o*_^*m*^, respectively. *a*_*o*_ and *b*_*o*_ are fixed parameters with *a*_*o*_>1 and *b*_*o*_>0. *m* and *n* are integers defining the scale and shift operations. In the special case of the dyadic transform, which is considered a particular type of DWT spectral analyzer, the parameters are typically set to *a*_*o*_=2 and *b*_*o*_=1. The DWT can be efficiently implemented using a multistage filter bank, where each stage involves downsampling the output of the low-pass filter. A practical implementation of this approach is presented in^10^, where the use of a DSP board (DSP1003) significantly reduced the execution time, enabling real-time processing capabilities.

**Fig. 6:**
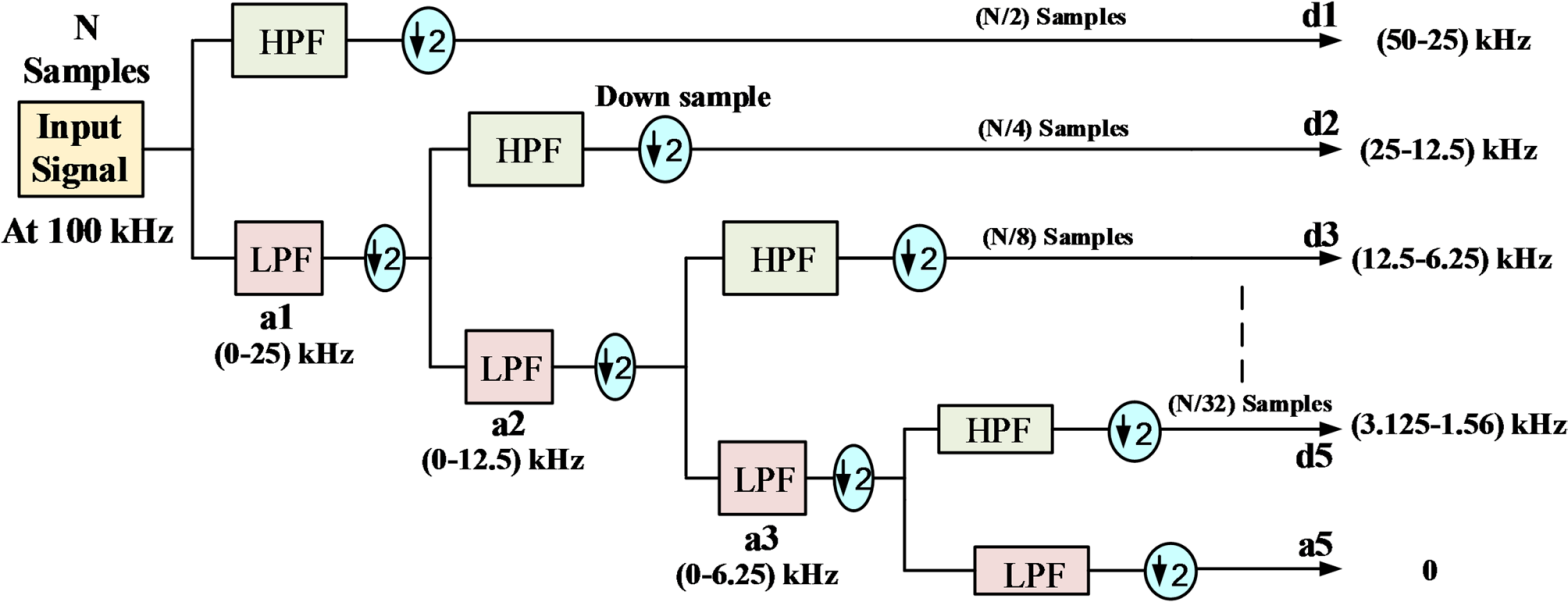
Implementation of DWT.

DWT utilizes filter banks to break down input signals into multiple levels of approximation and detail coefficients, as shown in Fig. 6. The performance of DWT, however, is highly dependent on the selection of the mother wavelet and decomposition depth. Various wavelet families, such as the Daubechies family, Bio-Orthogonals family, Coiflets family, and Symlet family, are evaluated for arc detection tasks in pantograph–catenary systems, as in this study.

To first explore wavelet-based arc detection in pantograph–catenary systems, the Daubechies 4 (db4) mother wavelet is selected for detailed analysis due to its widespread usage and proven effectiveness in power system disturbance detection^41^. The decomposition depth is fixed at six levels to ensure that the analysis captures the high-frequency transients and signal discontinuities associated with arc restriking. At the utilized sampling frequency, the sixth detail level (d6) corresponds to the 0.78–1.56 kHz band, while levels d4–d5 extend to higher frequency ranges. These bands lie beyond the dominant low-order harmonic spectrum of the traction system, thereby isolating non-stationary arc signatures from steady-state power-frequency components and background noise.

**Fig. 7:**
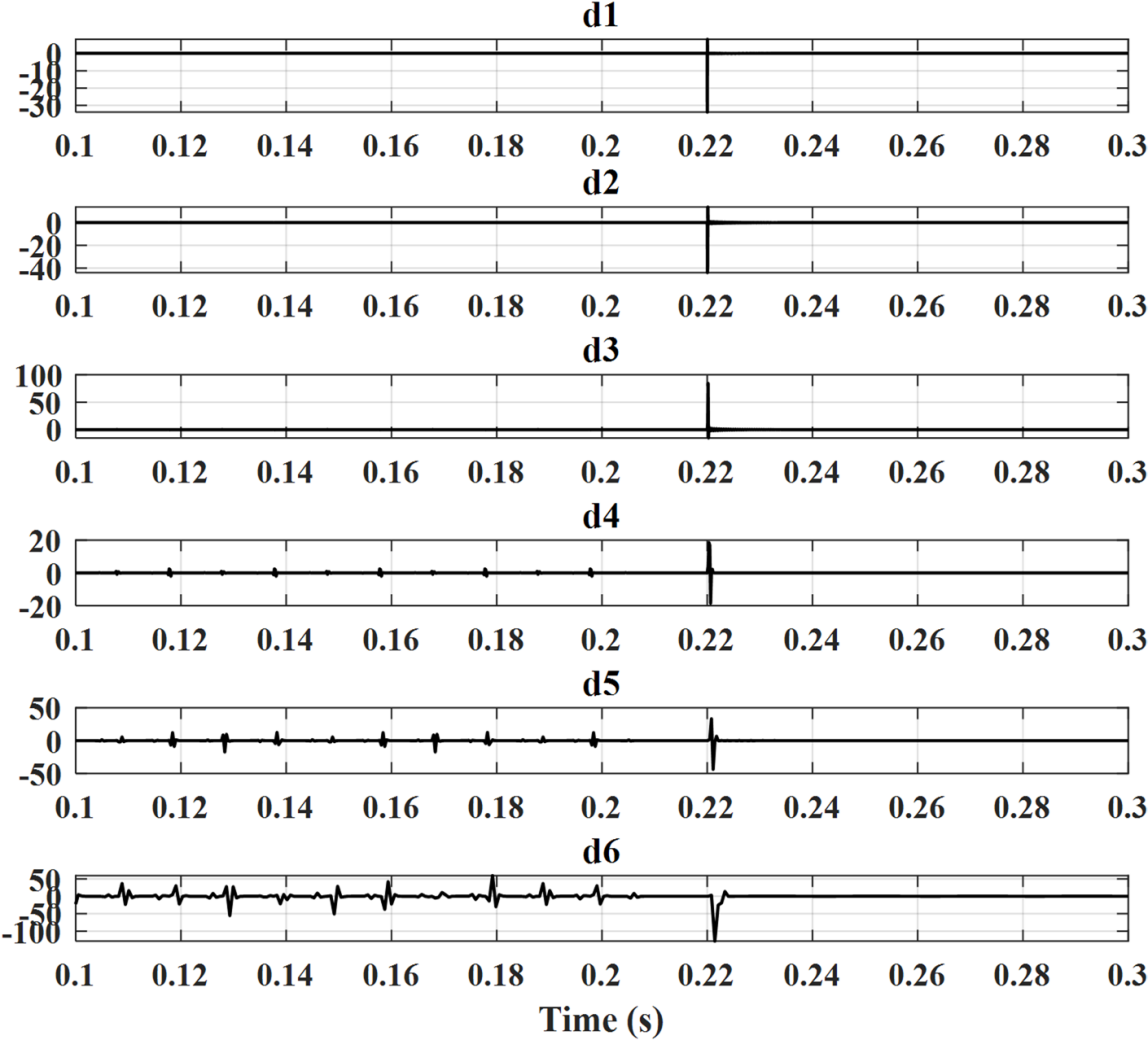
db4 details.

**Fig. 8:**
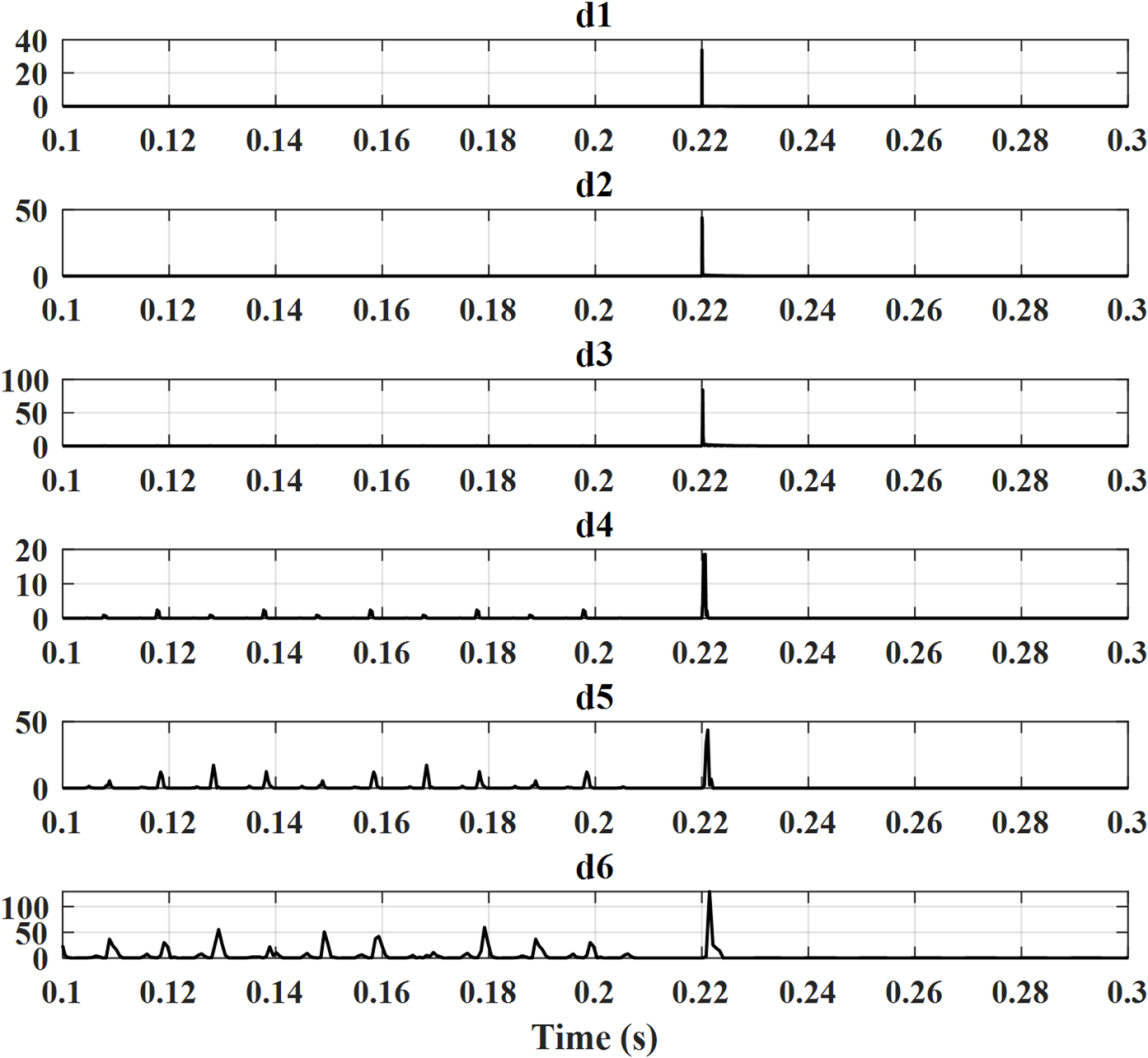
Absolute db4 details.

**Fig. 9:**
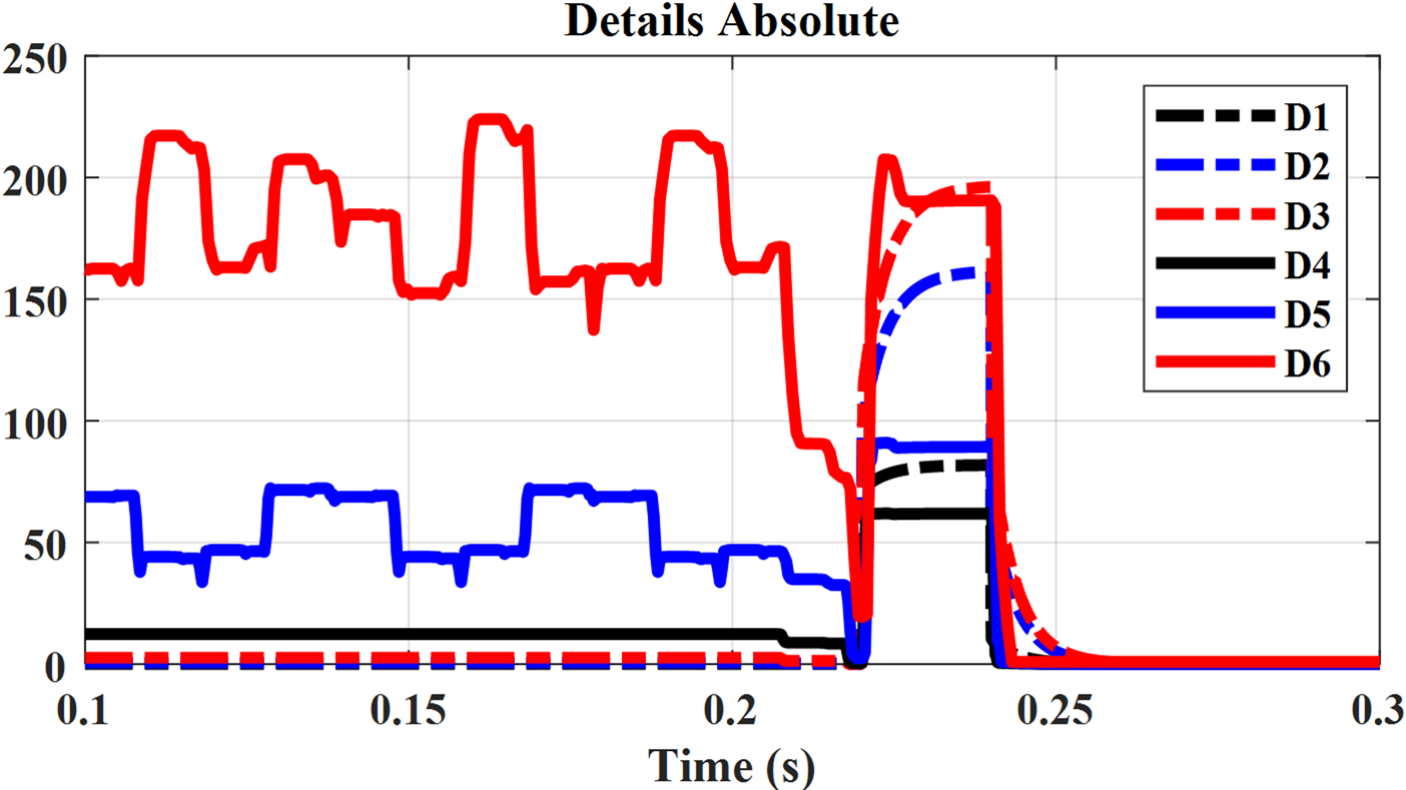
Absolute sum of power frequency sliding window of db4 details.

Figures 7 and 8 illustrate the corresponding detailed coefficient outputs obtained using the db4 wavelet. The analysis highlights that detail levels d4, d5, and d6 exhibit the highest magnitudes among all decomposition levels, particularly under arc fault conditions. This concentration of energy suggests that arc-related transients predominantly manifest within the mid-frequency bands captured at these levels. In Fig. 9, where the absolute sum of these details is evaluated over a power-frequency sliding window, these levels show a clear distinction between arc and normal states, indicating a strong correlation with the arc signature, especially in details level d4, d5, and d6.

Such significant magnitudes in d4, d5, and d6 details are critical for pattern recognition and serve as effective discriminative features. Therefore, these coefficients are considered highly suitable for input into a neural network classifier, as they encapsulate the transient arc characteristics with minimal noise interference. Their prominence affirms their utility in capturing the unique time–frequency patterns generated during arc events, thus enhancing classification accuracy and generalization capability in neural models.

Table 2 presents a comprehensive comparative analysis of different mother wavelets applied to the arc current signal of a pantograph–catenary system. It confirms that, across all mother wavelets examined, the maximum values of detail coefficients consistently occur at levels d4, d5, and d6. This observation supports the prior conclusion that these levels are the most relevant for arc feature extraction and should be prioritized in signal processing for machine learning applications.

While various wavelets were evaluated, it was observed that not all wavelets exhibit the same discriminative behavior. Lower-order wavelets typically produce elevated detail coefficients even under normal operating conditions, which may compromise their discriminative reliability. In contrast, higher-order wavelets such as db9 and Sym8 demonstrate a more selective and robust response, characterized by pronounced energy concentration during arc events and reduced sensitivity during normal operation. This behavior enhances the separability of arc-related features and provides a stronger basis for selecting suitable mother wavelets for reliable arc detection.

**Fig. 10:**
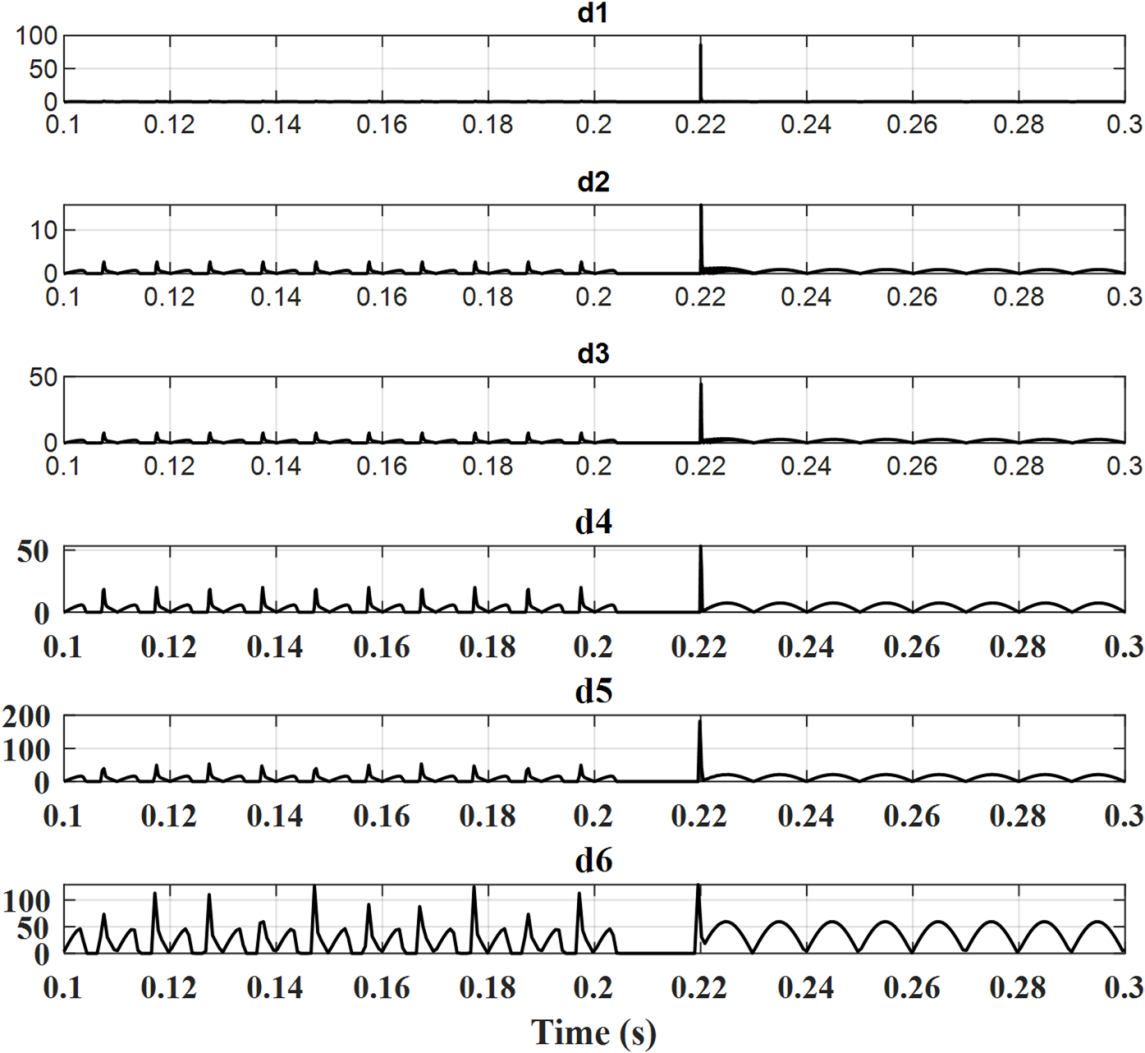
Absolute db1 details.

**Fig. 11:**
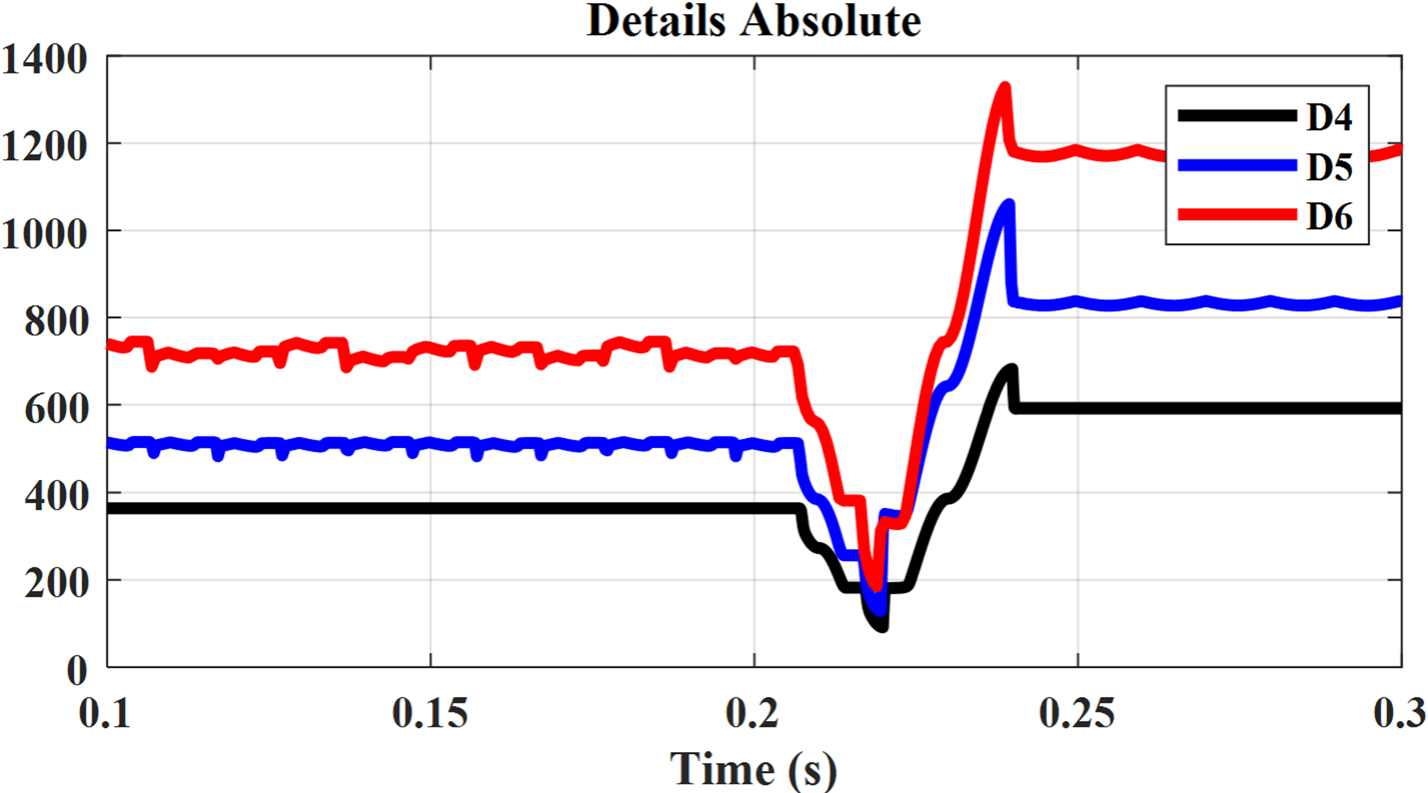
Absolute sum of power frequency sliding window of db1 details.

**Fig. 12:**
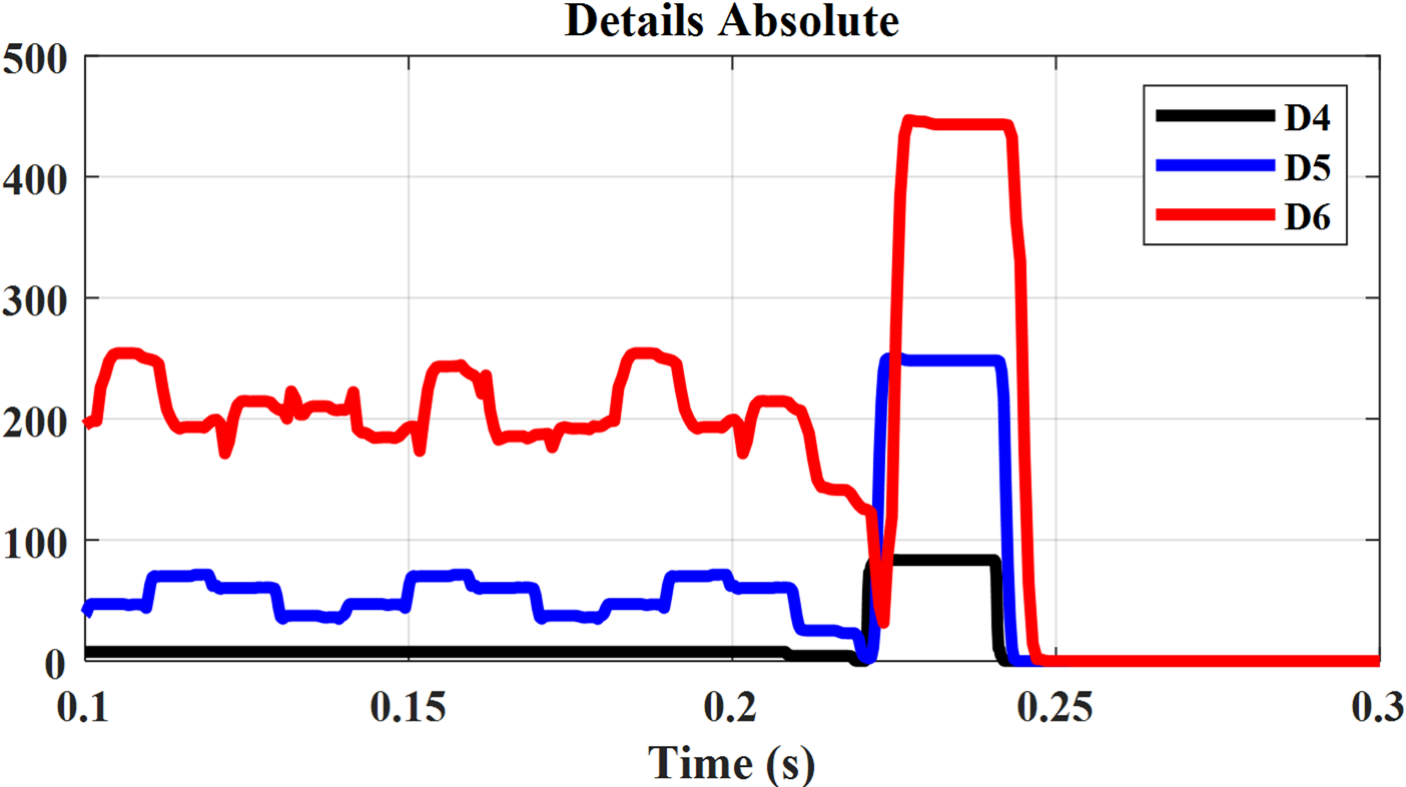
Absolute sum of power frequency sliding window of db9 details.

This behavior can be further observed when analyzing different wavelet families in detail. The Daubechies family demonstrates varied sensitivity across orders. While lower-order Daubechies wavelets db0 (Haar), db1, db2, and db3 show high values across all details (as indicated by strikethrough in Table 2). Unfortunately, there are high values for the details output of these Daubechies db0, db1, db2, and db3 during normal operation conditions (without series arcs). This phenomenon is evident in Figs. 10 and 11, making them less reliable for arc-specific detection. Therefore, this leads to false arc indication as the output is higher during non-arc intervals. Conversely, db6, db9, and db11 yield higher d4, d5, and d6 values specifically during arc periods, aligning better with the expected transient energy distribution. For example, Daubechies db11 reaches 263.92 at d6, and db9 peaks at 253.84, showing excellent sensitivity while maintaining robustness against normal waveform fluctuations. Therefore, db6, db9, and db11 are the most promising for arc feature extraction, and one of them will be selected for further neural network classification trials. Figure 12 shows the absolute sum of the details d4, d5, and d6 ascertained for the mother wavelet Daubechies db9 as an example.

**Table 2:** Comparative analysis of different mother wavelets.

Wavelet	d1	d2	d3	d4	d5	d6
db4	0.067	0.597	2.59	12.26	46.83	183.7
db5	0.02	0.38	4.04	9.114	52.27	207.8
db6	0.008	0.264	2.33	8.45	70.24	232.41
db7	0.003	0.183	3.601	8.24	56.59	230.17
db8	0.002	0.12	2.56	6.77	71.62	214.34
db9	0.001	0.082	3.08	7.59	71.36	253.84
db10	0.001	0.06	3.14	7.86	70.17	250.5
db11	0.001	0.05	2.39	7.56	65.85	263.92
db12	0.001	0.043	3.6	7.91	70.37	261.98
db13	0.001	0.03	1.7	7.1	72.49	268.5
db14	0.001	0.029	3.81	8.25	75.13	267.18
db15	0.001	0.026	1.158	7.39	59.06	240.75
db16	0.001	0.021	3.742	7.72	51.21	257
Bior 3.5	0.07	0.42	1.96	10.8	48.77	178.33
Bior 3.7	0.07	0.43	2.91	10.46	32.29	165.05
Bior 3.9	0.07	0.41	2.95	11.08	50.11	175.06
Bior 4.4	0.04	0.44	3.47	8.37	49.16	161.92
Bior 5.5	0.006	0.17	2.72	6.37	43.42	148.96
Bior 6.8	0.005	0.18	1.78	8.09	42.24	185.41
Coif2	0.05	0.57	3.23	11.34	66.06	196.71
Coif3	0.007	0.22	3.65	7.37	51.98	161.14
Coif4	0.001	0.12	2.32	7.71	49.69	192.66
Coif5	0.001	0.05	3.36	6.75	54.36	173.05
Sym4	0.06	0.63	4.43	11.13	66.1	185.86
Sym5	0.02	0.39	3.1	9.98	55.73	159.26
Sym6	0.008	0.25	3.69	8.19	47.92	188.07
Sym7	0.003	0.15	2.77	7.36	54.91	185.12
Sym8	0.002	0.12	2.08	7.98	57.52	197.2

For the Biorthogonal wavelets reported in Table 2, specifically bior3.5, bior3.7, bior3.9, bior4.4, bior5.5, and bior6.8, they achieved significantly high detail coefficients at d6. These wavelets show enhanced localization of arc transients while maintaining suppression during normal operation. Wavelets such as bior1.3, bior1.5, bior2.2, bior2.4, bior2.6, bior2.8, bior3.1, and bior3.3 are excluded due to high output during non-arc intervals, which contradicts the expected behavior for an effective arc detection tool and may cause false positives. The retained biorthogonal wavelets thus provide a more reliable and discriminative input for the neural network classifier.

In the Coiflet family, only Coif2, Coif3, Coif4, and Coif5 demonstrated acceptable sensitivity to arc-related components with consistently high values at d6 and relatively lower values during normal operation. Coif1, however, is excluded for the same reason as lower-order Daubechies and Biorthogonals. The Symlet family showed moderate performance, with Sym4, Sym5, Sym6, Sym7, and Sym8 retained due to their targeted energy concentration at d4, d5, and d6, particularly under arc scenarios. Sym2 and Sym3 is excluded due to the same reason mentioned above in normal operation.

Among the wide range of wavelet families evaluated using the results in Table II, the Daubechies family stands out, with the mother wavelets db6, db9, and db11, which show significant detail coefficient values during arc events while maintaining low output in normal conditions. Particularly, db9 offers a strong balance between sensitivity and robustness, making it a prime candidate for reliable arc detection. In contrast, the Biorthogonal, Coiflet, and Symlet families exhibited convergent results in their higher orders, with sym8 standing out for its effective energy concentration under arc conditions. Therefore, db9 and sym8 are selected to extract the transients generated due to series arcs of the pantograph-catenary electric system. This dual-wavelet approach enhances the reliability of decision-making by combining the strengths of two distinct wavelet families, ensuring greater generalization and robustness in neural network-based arc detection frameworks.

Since the proposed method performs binary classification (arc/no-arc), standard classification metrics, including accuracy, precision, recall, F1-score, and false positive rate (FPR) are used to comprehensively evaluate the model performance.

To further validate the selection of optimal mother wavelets, a quantitative classification performance comparison is conducted using representative candidates from different wavelet families. Table 3 summarizes the performance metrics of the ANN classifier when trained and tested using features extracted from each wavelet.

The results indicate that db9 achieves the highest classification accuracy 90.0% and F1-score of 0.846, along with a notably low false positive rate of 0.138, demonstrating excellent discrimination capability between arc and non-arc conditions. Similarly, Sym8 shows strong performance with an accuracy of 88% and F1-score of 0.82. In contrast, lower-order wavelets such as Haar exhibit significantly higher false positive rates, leading to poor overall classification performance. These findings quantitatively support the selection of db9 and Sym8 as the most suitable mother wavelets for reliable arc detection in pantograph–catenary systems.

In addition, the confusion matrix corresponding to the best-performing wavelet (db9) is presented in Table 4, providing a detailed representation of classification outcomes in terms of true positives (TP), false positives (FP), false negatives (FN), and true negatives (TN). The matrix confirms the robustness of the proposed method, achieving zero false negatives and a low false positive rate.

**Table 3:** Classification performance comparison across different mother wavelets.

Metrics	Mother Wavelet					
	haar	db4	db9	Bior 6.8	Sym5	Sym8
TP	11,001	11,001	11,001	11,001	11,001	11,001
FP	29,000	13,321	3998	13,513	13,449	4493
FN	0	0	0	0	0	0
TN	0	15,679	25,002	15,487	15,551	24,200
Accuracy	0.275	0.667	0.900	0.662	0.664	0.880
Precision	0.275	0.452	0.733	0.449	0.450	0.710
Recall	1.000	1.000	1.000	1.000	1.000	1.000
F1-score	0.431	0.623	0.846	0.620	0.621	0.820
FPR	1.000	0.459	0.138	0.446	0.464	0.15

**Table 4:** Confusion matrix of the proposed DWT–ANN model using db9 wavelet.

Actual: ARC	Predicted: ARC	Predicted: NON-ARC
	TP = 11,001	FN = 0
Actual: NON-ARC	FP = 3998	TN = 25,002

The confusion matrix of the best-performing wavelet (db9) demonstrates the strong classification capability of the proposed method, with zero false negatives and a low false positive rate.

## Artificial neural network decision

Arc phenomena in pantograph–catenary systems represent a critical concern in electric railway operations due to their potential to degrade equipment and disrupt power transmission. In this study, a machine learning approach based on artificial neural networks (ANNs) is developed to detect arc occurrences accurately and for real-time applications. The arc detection process relies on analyzing transient disturbances within the pantograph current signal, in which the transients are extracted using DWT^15^.

The detail coefficients at decomposition levels d4, d5, and d6 are selected for their ability to capture arc-related transients. These coefficients are normalized and used as input features for the neural network. Two mother wavelets—Daubechies 9 (db9) and Symlet 8 (sym8) are employed to evaluate their effectiveness in feature extraction.

**Fig. 13:**
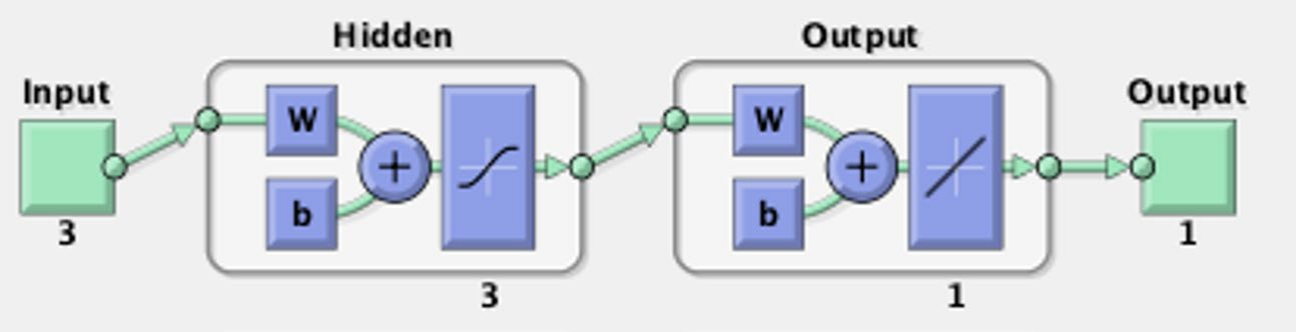
Used ANN Structure.

Figure 13 illustrates the corresponding architecture of the designed ANN used in this work. It shows a standard feedforward network consisting of an input layer of three input signals that are d4, d5, and d6 coefficients, one hidden layer with three neurons, and an output layer that provides a binary decision. The effectiveness of the three-neuron hidden layer is attributed to the DWT-based preprocessing applied prior to the classification stage. By utilizing the absolute sum of DWT detail coefficients followed by normalization, the transient patterns associated with arc events are clearly isolated. This high feature discriminability reduces the learning burden on the ANN, allowing a compact architecture to achieve reliable performance. Such a simplified configuration ensures low computational latency, making the proposed image-free method suitable for real-time pantograph–catenary monitoring, while remaining computationally more efficient than deep learning-based approaches.

**Fig. 14:**
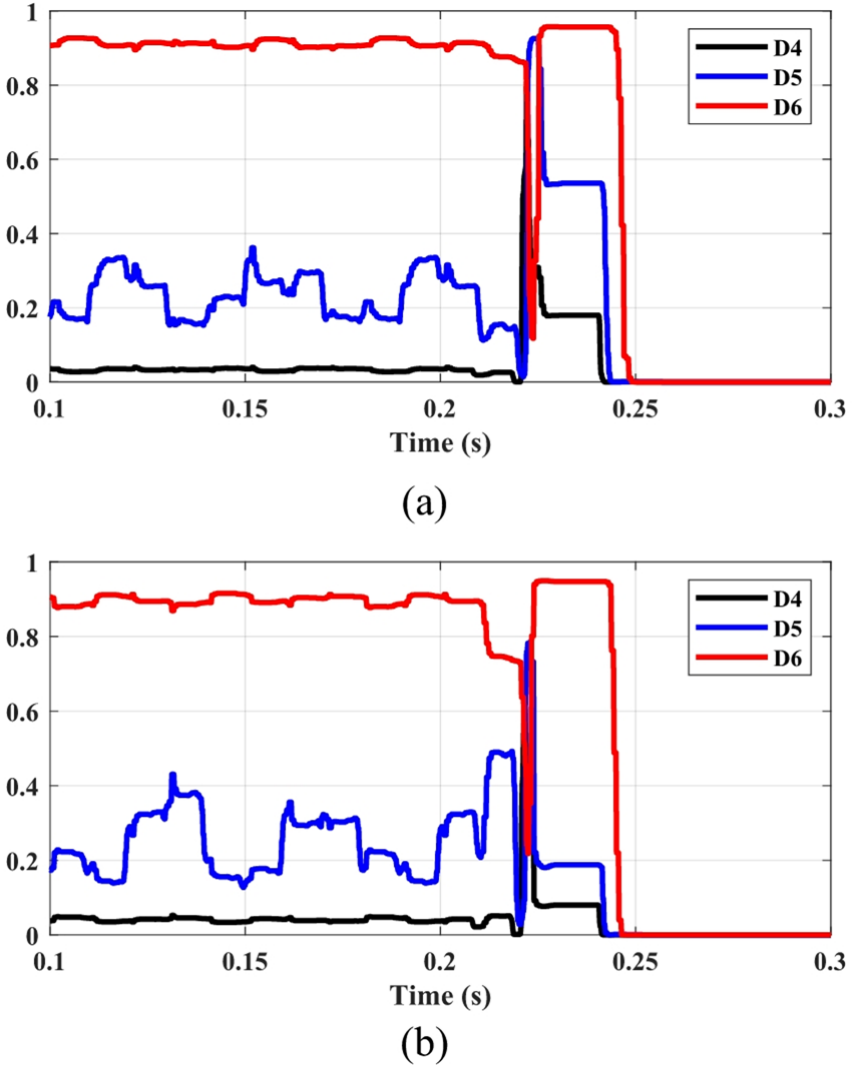
Normalized input data. (**a**) Daubechies db9 mother wavelet., (**b**) Sym8 mother wavelet.

This structure demonstrates a balance between simplicity and the capacity to learn arc patterns effectively. Figure 14 presents the normalized detail coefficients obtained from the db9, Sym8 wavelet. This preprocessing step helps scale the input data for better network performance. The figure confirms the feature range is controlled, enhancing network training stability.

The neural network is implemented in MATLAB as a feedforward network with a single hidden layer of three neurons using sigmoid activation. The Levenberg–Marquardt algorithm (trainlm) is used for training due to its fast convergence in nonlinear regression problems. The ANN performs binary classification to identify the presence (1) or absence (0) of arcs based on the extracted wavelet features. A total of 2,070 training samples were collected from simulation data, representing both arcing and non-arcing periods.

The real-time applicability of the proposed DWT–ANN framework is evaluated based on its computational efficiency. The DWT implementation relies on a sliding-window processing scheme, where the computational burden depends primarily on the length of the mother wavelet filters rather than the sampling frequency.

Experimental studies reported in the literature have demonstrated that multi-level DWT decomposition can be executed within tens of microseconds (e.g., 50–100 µs for five decomposition levels, depending on the wavelet type)^7^. In this work, the computational load is further reduced by limiting the decomposition levels and employing a compact ANN architecture. As a result, the total processing latency remains significantly shorter than the duration of a power cycle (20 ms at 50 Hz), indicating the potential for real-time operation.

**Fig. 15:**
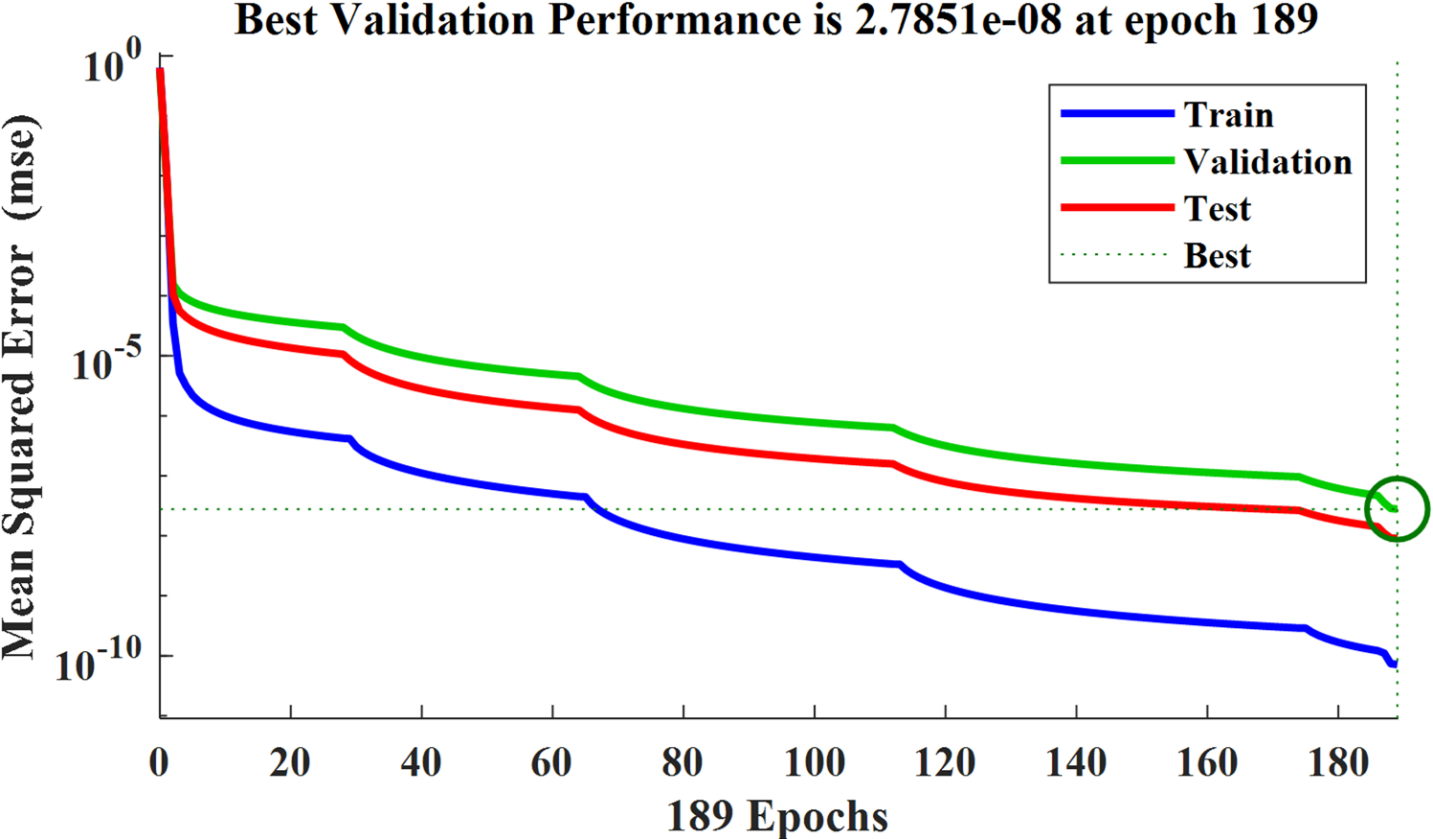
Mean Square Error (MSE) of the Designed ANN.

**Fig. 16:**
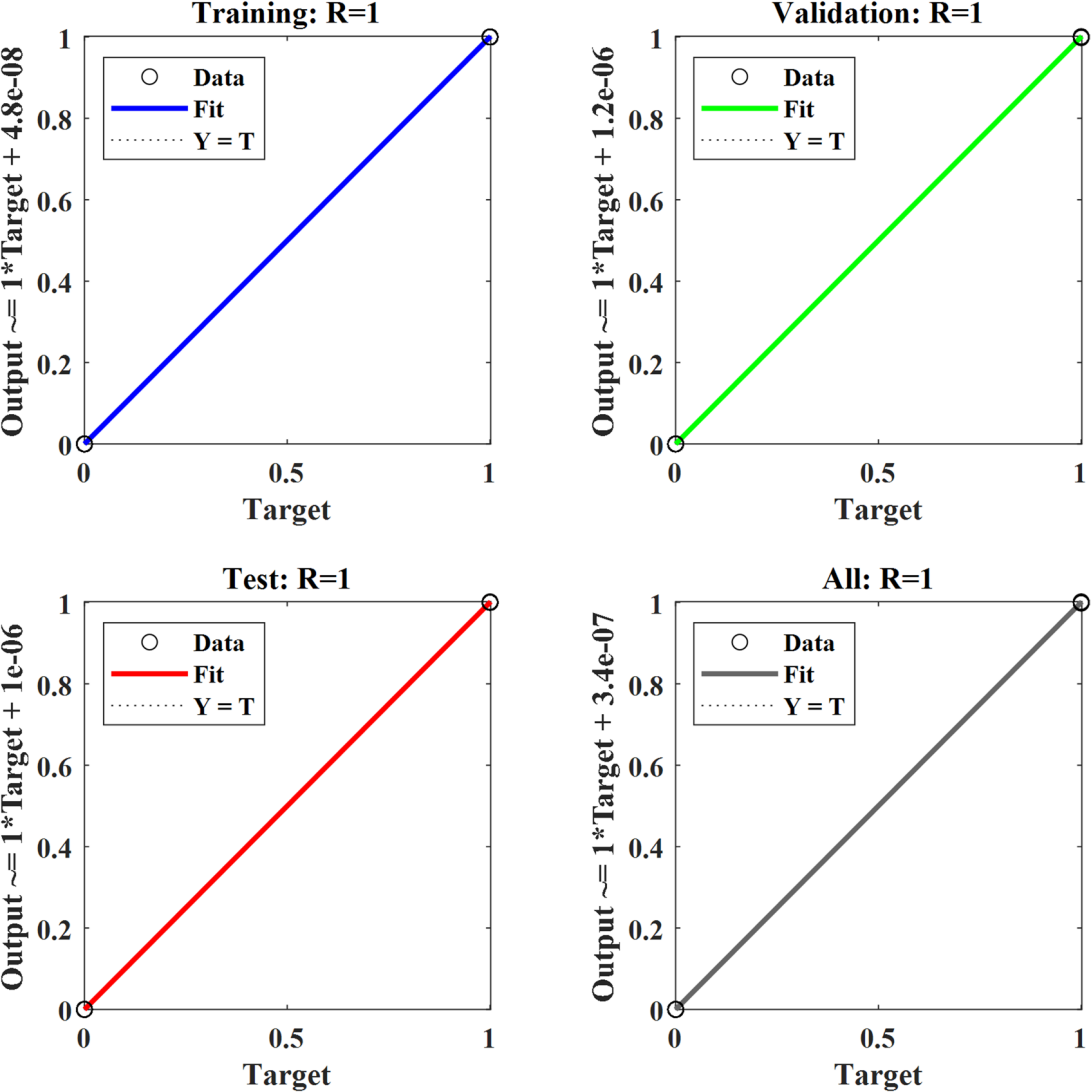
Regression Plot.

Figure 15 shows the mean square error during the training stage. A decreasing MSE curve indicates that the ANN is learning the relationship between input features and arc occurrence accurately. The smooth convergence without oscillations reflects a stable and effective training process. The corresponding error histogram results are created and evaluated. It is found that most errors are centered around zero, indicating that the network predictions closely match the expected outputs. This supports the model’s high classification accuracy. Also, the regression between network outputs and target values is evaluated as shown in Fig. 16. The closer the data points are to the diagonal line, the better the network performance. The regression results ensure high correlation visibility, confirming the ANN’s ability to accurately classify both arc and non-arc events.

**Fig. 17:**
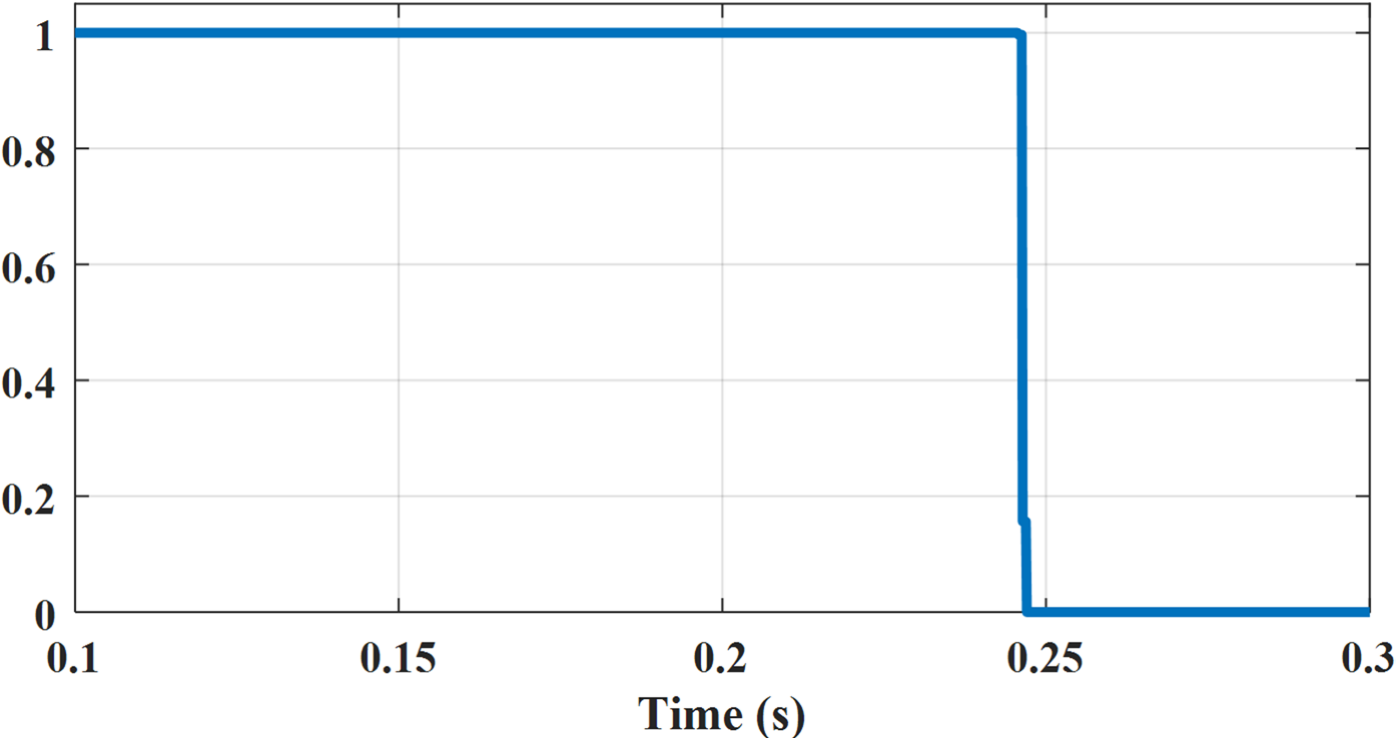
ANN Decision Output.

Figure 17 shows the final classification output of the ANN on test data. A clear separation between arc and non-arc predictions is observed. This validates the model’s ability to detect arc events in real time, making it suitable for practical deployment in railway monitoring systems.

## Algorithm robustness analysis

**Fig. 18:**
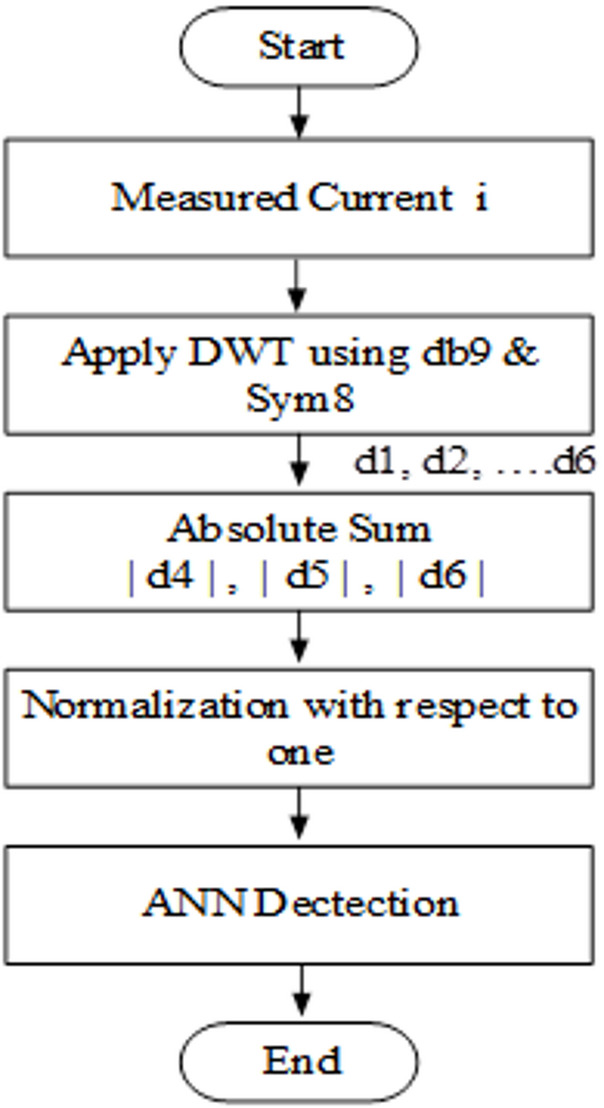
The Flowchart of the Proposed Arc Detection Algorithm.

In conclusion, the integration of DWT-based feature extraction with a compact ANN framework offers an effective and computationally efficient solution for detecting arc transients in railway systems. The approach demonstrates the potential of intelligent pattern recognition techniques in enhancing the safety and reliability of power collection infrastructures. The proposed algorithm for detecting the arc can be summarized by the flowchart as presented in Fig. 18.

To further validate the universality of the proposed algorithm, a generalized neural network model was developed using the features extracted from a single representative mother wavelet—Daubechies db4. This mother wavelet was chosen due to its balanced performance and well-established use in power system disturbance analysis. The network was trained solely on the normalized detail coefficients (d4, d5, and d6) derived from db4 decomposition. To evaluate its generalization capability, the trained neural network was subsequently tested using the features obtained from all other mother wavelets that were accepted in Table II, including various higher-order Daubechies, Biorthogonal, Coiflet, and Symlet functions. The network consistently delivered accurate arc classification across these diverse wavelet inputs, thereby demonstrating the algorithm’s robustness and universality.

This successful generalization is attributed primarily to the data normalization step applied before neural network training, which ensured that the input features maintained a consistent scale and dynamic range, independent of the specific wavelet used for feature extraction. Although the proposed framework demonstrates consistent performance across different wavelet families under simulation conditions, its performance under practical operating conditions may be influenced by measurement noise and parameter uncertainty. The use of the Discrete Wavelet Transform (DWT) provides partial robustness through its multi-resolution denoising capability, which helps separate high-frequency noise from transient arc-related features. In addition, the use of normalized energy-based features reduces sensitivity to variations in system parameters and signal scaling. Nevertheless, further validation using measured data is required to fully assess the generalization capability and practical robustness of the proposed method.

The observed performance of the proposed DWT–ANN framework can be explained by the inherent characteristics of arc signals and the adopted preprocessing strategy. Arc events are highly transient and non-stationary, characterized by rapid changes and high-frequency components during reignition periods, which makes conventional time-domain or frequency-domain analysis insufficient for accurate detection.

The use of DWT enables multi-resolution analysis, allowing the signal to be decomposed into different frequency bands where transient arc-related components can be effectively isolated. In particular, the selected detail levels capture high-frequency energy bursts associated with arc phenomena, while reducing the influence of background noise and steady-state components.

Moreover, the extraction of normalized energy-based features provides a compact and discriminative representation of arc behavior. This reduces the complexity of the learning task and allows the ANN model to focus on essential signal characteristics rather than raw data variations. As a result, the proposed framework achieves robust detection performance without requiring more complex or computationally intensive models. In this context, unlike traditional Fourier-based methods, which are limited in capturing the temporal localization of arc transients due to their inherent time–frequency resolution trade-off, the DWT–ANN approach maintains sensitivity to local signal fluctuations by focusing on specific detail levels where arc-related energy is concentrated.

To further examine the high classification performance, potential sources of overfitting were carefully considered. The discriminative nature of the extracted features, particularly those obtained through DWT-based preprocessing, contributes to clear separability between arc and non-arc conditions. Specifically, the extracted transient energy patterns are strongly associated with arc restriking events occurring after current zero crossings, which represent characteristic fingerprints of electric arcs. In addition, the use of a relatively simple ANN architecture helps limit model complexity and reduces the likelihood of overfitting. Furthermore, the variability in performance observed across different mother wavelets (as presented in Table III) indicates that the model performance is dependent on feature representation rather than being artificially inflated. Nevertheless, further validation using independent experimental datasets remains necessary to fully confirm the generalization capability of the proposed method.

In this study, although the ANN is trained using a single dataset, the evaluation is conducted over a wide range of arc conditions, including variations in arc time constant and stationary voltage, as well as different arc characteristics. Moreover, the use of DWT-based preprocessing enables the extraction of generalized transient features rather than reliance on raw signal patterns. This enhances the robustness of the proposed method. However, further validation using independent experimental datasets remains an important direction for future work.

This study presents a novel application of arc feature extraction and detection directly from the measured electrical current of the pantograph–catenary system, marking the first reported investigation to utilize traction current signals for arc detection in electric railway systems. As future work, the proposed DWT-based transient feature extraction and neural network detection scheme will be further investigated under varying stationary arc conductance and arc time constants. This will support the generalization and universality of the algorithm for broader real-world operating conditions.

## Conclusion

In this paper, the series arc transients in pantograph–catenary systems were successfully extracted using the DWT applied to measured traction currents, avoiding the need for image-based techniques. The pantograph arc behavior has been modelled using a simplified dynamic conductance model defining the stationary arc conductance by |*i|/U*_*st*_ with *U*_*st*_ = 2.3 kV. The comparative analysis of various mother wavelets applied to arc current signals in a pantograph–catenary system revealed that the detail levels d4, d5, and d6 consistently exhibit the highest sensitivity to arc transients. Among all tested wavelets, Daubechies db6, db9, and db11 emerged as the most effective, with db9 demonstrating the optimal balance between sensitivity to arc-induced energy and robustness against normal operation and achieving a peak of 253.84 at d6. Similarly, higher-order Biorthogonal (e.g., bior3.5, bior3.9), Coiflet (e.g., Coif4, Coif5), and Symlet (e.g., sym8) wavelets showed improved arc localization with suppressed response during non-fault intervals. Conversely, several mother wavelets produced high detail coefficients even in the absence of arc faults, providing undesirable behavior under normal, non-arc conditions. They were such as lower-order Daubechies db0, db1, db2, and db3, Biorthogonal bior1.3, bior1.5, bior2.2, and bior2.4, Coif1, Sym2, and Sym3 exhibited elevated energy levels during steady-state operation. These wavelets, though responsive, lack the specificity required for reliable arc detection.

Daubechies db9 and Symlet sym8 are selected as the optimal mother wavelets for arc feature extraction, combining high sensitivity to transient arc signals with minimal response to normal operation. This dual-wavelet strategy ensures enhanced reliability and generalization for integration into machine learning-based arc detection systems. Compact ANN with a single hidden layer (3 neurons) trained by Levenberg–Marquardt achieves rapid convergence and low mean-square error. The 2,070 simulated pantograph-current samples yield a regression coefficient ≈ of 0.998, confirming excellent generalization. The proposed algorithm showed promising behavior in accurately detecting pantograph arcs and proved to be highly reliable across a wide range of mother wavelet families, reinforcing its robustness and practical applicability.

Despite the promising results, this study is primarily based on simulation data. Future work will focus on experimental validation by developing a hardware-in-the-loop (HIL) setup and using field-measured signals. These steps will help assess the algorithm’s robustness under noise and varying operating conditions in practical railway systems.

## Data Availability

The datasets used and/or analyzed during the current study are available from the corresponding author on reasonable request.
